# Adaptive Servo-Ventilation as a Novel Therapeutic Strategy for Chronic Heart Failure

**DOI:** 10.3390/jcm11030539

**Published:** 2022-01-21

**Authors:** Teruhiko Imamura, Nikhil Narang, Koichiro Kinugawa

**Affiliations:** 1 Second Department of Internal Medicine, University of Toyama, 2630 Sugitani, Toyama 930-0194, Japan; kinugawa-tky@umin.ac.jp; 2 Advocate Christ Medical Center, Oak Lawn, IL 60453, USA; nikhil.narang@gmail.com

**Keywords:** heart failure, hemodynamics, congestion

## Abstract

The introduction of new therapeutics for patients with chronic heart failure, including sacubitril/valsartan, sodium-glucose cotransporter 2 inhibitors, and ivabradine, in addition to beta-blockers, angiotensin converting enzyme inhibitors, and mineralocorticoid receptor antagonists, lends an opportunity for significant clinical risk reduction compared to what was available just one decade ago. Further clinical options are needed, however, for patients with residual clinical congestion refractory to these therapies. Adaptive servo-ventilation is a novel therapeutic option to address significant clinical volume in cases resistant to medical therapy. The aggregate benefit of these additional therapeutic strategies in addition to foundational medical therapy may be a promising option in the selected candidates who do not achieve acceptable clinical and quality-of-life improvements with oral medical therapy alone. Now is the era to reconsider the implication of an adaptive servo-ventilation-therapy-incorporated medical therapeutic strategy for patients with congestive heart failure.

## 1. Introduction

Several novel therapies have been introduced over the last decade that both improve quality of life and reduce mortality in patients with chronic heart failure, including sacubitril/valsartan (ARNI), sodium-glucose cotransporter 2 (SGLT2) inhibitor, and ivabradine [1]. Up-titration of neurohormonal agents including beta-blockers and mineralocorticoid receptor antagonists to maximal doses in addition to these new therapies is essential to achieve the best clinical benefit [2]. The additional risk reduction for heart failure hospitalization or death with contemporary four-tier guideline-directed medical therapy (ARNI, beta-blocker, mineralocorticoid inhibitor, and SGLT2i) compared to angiotensin-converting enzyme inhibitors and beta-blockers alone is >50%. The guidelines of the Japanese Circulation Society recently published a focused update to emphasize the importance of these life-saving therapies, with a clear recommendation of urgency to rapidly up-titrate these therapies to doses specified in respective landmark clinical trials [3].

In addition to the survival benefit, improvement in patient-reported outcomes including functional status and quality of life is also of paramount importance [4]. Much of this is related to the treatment of congestion. Although these new medical therapies significantly reduce the burden of congestions, some patients suffer from residual volume overload that considerably reduces functional capacity. Furthermore, adequate decongestion at index discharge following heart failure hospitalization is unsurprisingly strongly associated with clinical outcomes [5]. Loop diuretics are conventional tools to treat pulmonary and systemic congestion [6]. Tolvaptan, vasopressin type-2 receptor antagonist, is a potent natriuretic agent that has been utilized for a decade [7]. Tolvaptan as a diuretic therapy improves pulmonary/systemic congestion while not worsening renal function [8]. The cost of this medication in addition to lack of evidence when combined with contemporary heart failure therapies are limiting factors to justify the widespread implementation of tolvaptan. There remains a gap in care for patients with residual congestion, for which nonmedical therapeutic strategies should be considered.

**Figure 1:**
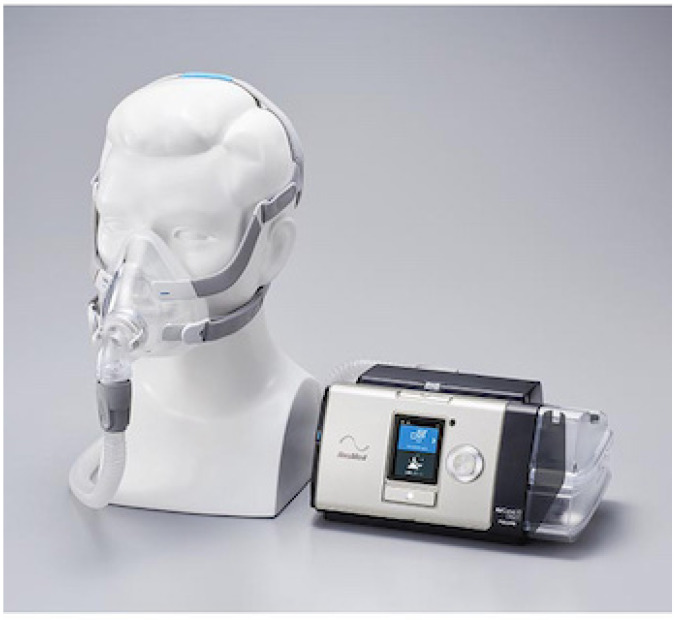
Adaptive servo-ventilation device set.

Adaptive servo-ventilation (ASV; AutoSet-CS; ResMed, Sydney, Australia) is a noninvasive, positive pressure ventilation tool that reduces work of breathing, suppresses sympathetic nervous system activation, and improves pulmonary/systemic congestion via decreasing cardiac preload and afterload in patients with congestive heart failure, if appropriately utilized at least for 4 h during the night (Figure 1) [9]. Importantly, this occurs irrespective of the existence of sleep-disordered breathing [10]. Although large-scale randomized control trials of ASV in patients with chronic heart failure did not demonstrate a mortality benefit [11], ASV is still being utilized with success in some scenarios to improve patient symptomology [12].

We believe that ASV therapy can be an effective strategy in managing persistent pulmonary/systemic congestion refractory to medical treatment [13], and should be reconsidered as part of the therapeutic armamentarium in patients with chronic heart failure, even in the era when novel medical agents have been introduced, if appropriately utilized as discussed in this review.

## 2. Management of Congestion in the Current Era

Adequate control of congestion is a critically important goal in chronic heart failure management to both reduce mortality and morbidity and improve patients’ symptomology and quality of life [14]. Furthermore, residual pulmonary congestion misdiagnosed by clinical assessment at index discharge is associated with worse clinical outcomes [5].

Sacubitril/valsartan was the first of the new medical therapies in patients with chronic heart failure shown to reduce mortality and morbidity compared to the enalapril arm in the PARADIGM-HF trial [15]. However, the secondary analysis demonstrated reduced efficacy of sacubitril/valsartan in patients with multiple signs of congestion based on clinical exam [16]. SGLT2 inhibitors have pleiotropic benefits with no single direct mechanism to explain the substantial clinical benefit in patients with heart failure and reduced ejection fraction. Furthermore, there is a longitudinal benefit in patients with chronic kidney disease with and without heart failure, which is unique among the current heart failure-specific therapies [17]. However, the renoprotective effect of SGLT2 inhibitors was less expected in patients with insufficient cardiac unloading, indicated as higher plasma B-type natriuretic peptide levels [18]. The additive effects of these novel medical therapies may potentiate clinical benefit with alternative therapies such as ASV, whereas this benefit was not realized in prior clinical trials before the debut of both ARNI and SGLT2 inhibitor therapy.

In the acute phase of decompensated heart failure, early de novo administration of beta-blocker therapy is generally contraindicated given the risk of worsening cardiac output due to negative inotropic effects in the presence of pulmonary congestion [19]. This may be a time point when ASV can be utilized to more immediately alleviate congestion and thus shorten the period to initiation of foundational medical therapy [20].

## 3. Adjustment of Adaptive Servo-Ventilation Therapy

The indications, pressure settings, and timing of termination need to be identified during ASV therapy. For successful ASV therapy and avoidance of congestion, the baseline existence of pulmonary congestion is necessary to be confirmed [13]. Inappropriate ASV therapy for those without pulmonary/systemic congestion would rather decrease cardiac output, deteriorate hemodynamics, and increase cardiovascular mortality and morbidity.

**Figure 2:**
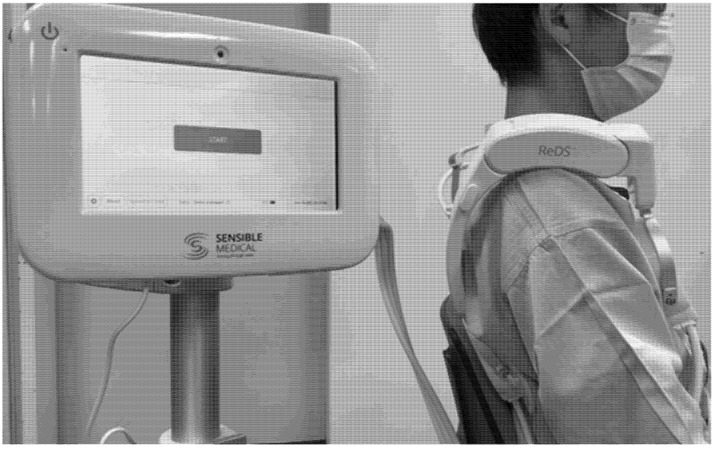
A monitor and a sensor of the remote dielectric sensing system.

Recently, remote dielectric sensing (ReDS^TM^, Sensible Medical Innovations Ltd., Netanya, Israel) system, which is a noninvasive electromagnetic-based technology to quantify lung fluid volume, has been introduced in the clinical setting (Figure 2) [21]. Prior work has demonstrated the correlation between ReDS value and lung fluid level measured by high-resolution computed tomography [22,23]. The ReDS system is a promising tool to accurately assess for pulmonary congestion and may be an appropriate precursor modality to screen for patients that may benefit from early ASV therapy when decompensated heart failure is suspected.

**Figure 3:**
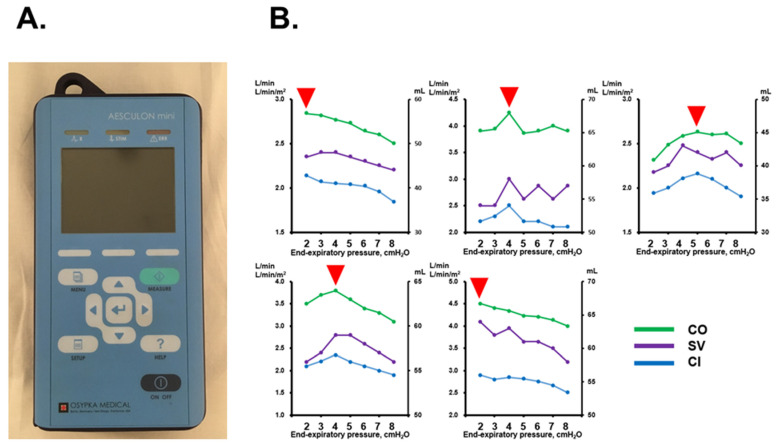
ASESCULON mini device (**A**) and examples of pressure ramp test (**B**) [25]. Red arrow heads indicate end-expiratory pressures with maximum cardiac output. CO, cardiac output; SV, stroke volume; CI, cardiac index.

Another novel tool in the care of heart failure patients is the AESCULON mini^TM^ (Osypka Medical, Berlin, Germany), which noninvasively estimates cardiac output (Figure 3) [24]. With ASV therapy, inappropriately high pressure settings may rather lower cardiac output during the ASV therapy [12]. We thus propose a pressure ramp test, during which cardiac output is measured at each pressure setting to optimize end-expiratory pressure to accompany maximum cardiac output (Figure 3) [25]. ReDS values might also be measured during the pressure ramp test to assess lung fluid levels at each pressure setting [26].

Continuation of ASV after improvement in clinical congestion is not encouraged and may explain the lack of superior clinical benefit in the SAVIOR-C randomized control trial [10]. However, optimal methodologies for clinicians deciding the appropriate timing to terminate ASV therapy remain unestablished. Monitoring of daily congestion through utilization of the ReDS system, in addition to congestion-related biomarkers including adrenomedullin, should allow the clinicians to accurately tailor the use of ASV. When ReDS value trends to decrease, we should consider terminating the ASV therapy to avoid hemodynamic deterioration.

## 4. Renoprotection

A cardiorenal syndrome is an additional clinical syndrome within heart failure presentations which is independently associated with worse clinical outcomes [27]. Unoptimized chronic heart failure can lead to renal congestion, decreased renal perfusion and further downstream activation of inflammatory and maladaptive neurohormonal pathways. Renal impairment worsens volume overload, subsequentially increasing cardiovascular preload and afterload.

Up-titration of loop diuretics is one option to manage volume overload triggered by progressive chronic kidney disease. However, high-dose diuretics are associated with inappropriate stimulation of the renin–angiotensin system, and may further worsen renal function [4]. Angiotensin-converting enzyme inhibitor, ARNIs, and mineralocorticoid receptor antagonist may increase serum creatinine and potassium levels particularly in elderly patients with chronic kidney disease [28,29].

ASV therapy might be a promising alternative to manage congestion while maintaining renal function. We recently demonstrated that ASV therapy maintained renal function by comparison with the pre-ASV treatment period (i.e., pretreatment versus on-treatment) [30]. Underlying mechanisms should be multifactorial. Improvement of cardiac output following the initiation of ASV supports would enhance renal perfusion and ameliorate renal ischemia. Suppression of sympathetic nerve activity by respiratory stabilization would dilate the renal artery and maintain renal perfusion, as well as prevent the progression of renal tissue apoptosis and fibrosis. Of note, those who achieved a reduction in diuretics dosage had greater long-term renal function preservation. Thus, ASV therapy might have a direct and indirect protective effect on kidney function.

Other therapies may also have the potential to preserve kidney function. Tolvaptan, a vasopressin type-2 receptor antagonist, may have a neutral impact on renal function as opposed to conventional loop diuretics. Tolvaptan has potent aquaretic properties and may decrease the dosage needed of typical loop diuretics, though in some countries this presents a cost challenge [8]. SGLT2 inhibitors, in addition to being associated with significant clinical benefits in patients with chronic heart failure, have also been shown to have considerable renoprotective effects in a large-scale randomized control trial [17]. One of the proposed mechanisms is the regulation of tubularglomerular feedback. Although further studies are needed, sacubitril/valsartan might also slow the worsening of renal function through an improvement in ventricular function and lowering the volume and pressure burden experienced by kidneys [31]. Although categorized as a mineralocorticoid receptor antagonist, the newly introduced esaxerenone might reduce proteinuria and better preserve renal function [32]. Another nonsteroidal, selective mineralocorticoid receptor antagonist, finerenone, may also suppress progression of chronic kidney disease and prevent cardiovascular events [33]. The combination of these heart failure therapies with ASV may be a promising strategy to manage cardiorenal syndrome.

## 5. Conclusions

In the current era with a new regimen of available medical therapies for heart failure, a combination of ASV and medical therapy may be a promising option for patients with considerable congestion and impaired renal function.

## Data Availability

Data are available on appropriate requests.
